# Community-based navigators for tobacco cessation treatment: a proof-of-concept pilot study among low-income smokers

**DOI:** 10.1186/s12889-015-1962-4

**Published:** 2015-07-09

**Authors:** Arnold H. Levinson, Patricia Valverde, Kathleen Garrett, Michele Kimminau, Emily K. Burns, Karen Albright, Debra Flynn

**Affiliations:** 1 Department of Community & Behavioral Health, Colorado School of Public Health, Aurora, CO USA; 2 University of Colorado Cancer Center, Mail Stop F542, 13001 East 17th Place, 80045 Aurora, CO USA; 3 Mercy Family Medicine, Mercy Regional Medical Center, Centura Health, Durango, CO USA; 4 Head Start, City of Oakland, CA USA

## Abstract

**Background:**

A majority of continuing smokers in the United States are socioeconomically disadvantaged (SED) adults, who are less likely than others to achieve and maintain abstinence despite comparable quit-attempt rates. A national research initiative seeks effective new strategies for increasing successful smoking cessation outcomes among SED populations. There is evidence that chronic and acute stressors may interfere with SED smokers who try to quit on their own. Patient navigators have been effectively used to improve adherence to chronic disease treatment. We designed and have pilot-tested an innovative, non-clinical community-based intervention – smoking cessation treatment navigators – to determine feasibility (acceptance, adherence, and uncontrolled results) for evaluation by randomized controlled trial (RCT).

**Methods:**

The intervention was developed for smokers among parents and other household members of inner city pre-school for low-income children. Smoking cessation treatment navigators were trained and deployed to help participants choose and adhere to evidence-based cessation treatment (EBCT). Navigators provided empathy, resource-linking, problem-solving, and motivational reinforcement. Measures included rates of study follow-up completion, EBCT utilization, navigation participation, perceived intervention quality, 7-day point abstinence and longest abstinence at three months. Both complete-case and intent-to-treat analyses were performed.

**Results:**

Eighty-five percent of study participants (n = 40) completed final data collection. More than half (53 %) enrolled in a telephone quitline and nearly three-fourths (71 %) initiated nicotine replacement therapy. Participants completed a mean 3.4 navigation sessions (mean 30 min duration) and gave the intervention very high quality and satisfaction ratings. Self-reported abstinence was comparable to rates for evidence-based cessation strategies (21 % among study completers, 18 % using intent-to-treat analysis; median 21 days abstinent among relapsers).

**Conclusions:**

The pilot results suggest that smoking cessation treatment navigators are feasible to study in community settings and are well-accepted for increasing use of EBCT among low-income smokers. Randomized controlled trial for efficacy is warranted.

## Background

After decades of unequal progress in combating the smoking epidemic, a majority of continuing smokers in the United States are socioeconomically disadvantaged (SED) [1–11]. Many studies [12–19], although not all [20, 21], have found SED smokers as likely or more likely than other smokers to try to quit. The disparity in smoking prevalence persists because SED quit-attempters are less likely to achieve and maintain abstinence [4, 9, 13, 22, 23]. Leading suspected factors include underuse of evidence-based treatment (EBT) [22, 24–27], and chronic exposure to psychosocial stressors without adequate social support or healthy coping strategies. [27–29] A national research initiative seeks effective new strategies for increasing successful smoking cessation outcomes among SED populations [30].

A fair amount of evidence suggests that social position moderates exposure to acute life stressors, chronic stress, and the acquisition of healthy coping strategies [31–34]. Individuals faced with exogenous stressors generally engage in coping behaviors to mitigate the experience, and specific stress-response behaviors are chosen and shaped by social-environmental characteristics [35]. Smoking and other unhealthy self-soothing behaviors may represent attempts to cope with the stressors of daily life [36]. When SED smokers experience acute or chronic stress, they may be less concerned with long-term ill-health effects of smoking than with a perceived need for short-term relief of negative moods and the momentary relaxation they associate with smoking. To quit smoking, one not only foregoes self-soothing effects but also experiences added stressors of nicotine withdrawal and cravings. Further, although guideline-based cessation methods offer the best chance of quitting, the treatments impose their own demands. Treatment-assisted quit-attempters must choose, obtain, refill, and adhere to pharmacotherapy; schedule and attend counseling sessions in person or by telephone; and create or choose, then repeatedly carry out, planned behaviors (quit date, trigger- and cue-avoidance, healthy replacement activities, treatment activities, *etc.*). Most smokers find quitting very difficult. SED may make it even harder for quit-attempters to keep cessation counseling appointments, pay for medication refills, and avoid or recover from lapses to smoking.

### Intervention model

Patient navigation is an individualized intervention to identify and address patient-level barriers to health care [37], especially to improve timeliness and completeness of care [38]. The model was developed in response to health care disparities attributable to complex and fragmented health care systems, cultural incongruence, and unequal access to preventive and treatment services [39]. The navigation role applies skills used in case management, social work, and community health work, and operates by assessing and addressing needs, barriers, cultural health beliefs, social and emotional support and health literacy. Although navigators respond to issues as they arise [40], recent applications show they can also provide anticipatory guidance that prevents barriers from arising [41]. The navigator-patient relationship is ideally maintained throughout the treatment process.

Patient navigation is efficacious in improving timeliness and adherence to cancer screening guidelines and diagnostic workups after abnormal screening [38, 42]. Systematic reviews conclude that navigators improve cancer screening rates and reduce delays in diagnosis [38, 43]. The Patient Navigator Research Program (PNRP), funded by the National Cancer Institute, found that patient navigators increased adherence to cancer diagnostic procedures [43–47], although the impact on treatment-timing was inconclusive [43, 48, 49]. Two PNRP sites that used randomized controlled trial (RCT) designs found that patient navigation significantly increased satisfaction with care among some disadvantaged groups [49].

Although patient navigators commonly operate in clinical settings, they are increasingly deployed in community settings, *e.g.*, to work with stroke survivors [50] or individuals with chronic diseases [51], to conduct screening outreach [52], to link patients with health system navigators [53], and to provide culturally informed guidance [54, 55].

### Communication style: motivational interviewing

Motivation to quit smoking is highly labile and can fluctuate or vacillate quickly and frequently [56, 57]. It has been called a “bistable” phenomenon [58] because it is unstable during an indeterminate period of transition from smoking to nonsmoking but can settle at either valence, *i.e.*, prolonged abstinence or relapse. Similarly, “relapse proneness” has been proposed as a heuristic investigational model in which several fluctuating forces may exceed a threshold and overwhelm motivation to stay abstinent [58]. Put most simply, cessation is a process, not an event, and is characterized by volatile opposing desires – to be done with smoking and to smoke another cigarette.

Strengthening motivation is a primary objective of motivational interviewing (MI), a brief, directive client-centered counseling intervention [59, 60]. MI applies a set of therapeutic techniques that identify barriers to and ambivalence about change. An MI provider works collaboratively and empathically to resolve ambivalence and assist the client to develop tailored behavioral goals. Meta-analyses have found strong empirical support for MI across a multitude of addictive behaviors [61], and MI is recommended for increasing future smoking cessation attempts [62]. Two meta-analyses [62, 63] have found MI effective for smoking cessation compared to no-treatment controls, written materials, and nonspecific treatment as usual.

In the larger of the two MI meta-analyses [63], the effect size against weak comparators was significantly larger among African Americans than among whites; at the same time, an RCT among African American light smokers found MI ineffective while a strong comparator – health education – was effective [64]. Among Hispanics, MI’s meta-analytic effect size was equivalent to the effect sizes of both weak and strong comparators [63]. Although few studies have examined MI efficacy among SED populations, an RCT among a racially and ethnically diverse low-income smoking population [65, 66] found significantly lower carbon monoxide and household nicotine levels among MI participants at 3- and 6-month follow-up.

### SED and power differentials

Like other professions, health care professions are social constructions that are based on a hierarchy of knowledge and which signal the presence of authoritative knowledge [67]. In contrast, SED by definition connotes social disempowerment. With few exceptions [68, 69], studies have found significantly lower trust of medical providers and the health care system among low-income and ethnic minority populations [70–75]. In the presence of higher authority, SED individuals may experience feelings of blame, shame, or condescension, especially if the interaction involves the individual’s (health) behavior.

Lay status may allow interveners to reduce or eliminate hierarchy and mistrust. We chose to employ lay navigators to avoid any distancing effects attributable to patient-provider and social worker-client relations. Lay navigators might conceivably be less effective than those with professional training, but our team has not encountered this problem, and limited available evidence does not support it. A study of peer vs. master’s-level outreach workers found no difference in protocol fidelity, quality or outcomes during and after delivery of an MI-based intervention to retain adolescents and young adults in HIV care [76]. The larger meta-analysis of MI found no negative effect associated with a lack of credential or profession, although the authors note the paucity of studies. MI co-founder William Miller asserts that a helping professional’s ability to empathize with clients matters more than the professional’s training background [63].

We developed and pilot-tested a navigator-like intervention to help SED smokers engage with and adhere to evidence-based smoking cessation treatment, using a patient-centered communications style delivered by lay navigators in a community setting serving SED smokers. The intention was to offset SED-related deficits that may make smoking cessation more difficult for SED smokers. The current article describes the intervention’s development, results of a proof-of-concept feasibility study (R21CA141569), and directions for further research.

## Methods

The research team included a behavioral scientist (AL), a preventive medicine physician (EB), a project manager (MK), the community site’s health specialist (DF), a counselor with national MI training credentials (KG), and a patient navigation trainer and curriculum developer (PV). Two community members served as volunteer study advisers. The study protocol and consent materials and processes were approved by the Colorado Multiple Institutional Review Board; written consent was obtained before enrollment was allowed.

### Study site

The federal Head Start program [77] serves low-income children and requires considerable involvement from parents and caregivers. With assistance from the City and County of Denver, we initiated a research collaboration with the largest preschool in Denver’s Great Kids Head Start program. A convenience-sample survey of parents at the site (*n* = 54) found 52 % of households had one or more current smokers, and 82 % of respondents from smoker households said they and/or another smoking householder would be very or somewhat likely to try using smoking cessation support if it were offered at the Head Start preschool.

### Intervention overview

We designed the intervention, *smoking cessation treatment navigation* (SCTN), to reflect principles of patient navigation: Complement rather than replace existing treatment resources; provide tailored guidance for overcoming treatment barriers; solve problems as they arise, and provide non-clinical emotional and social support and motivational reinforcement during the cessation process. The objective was to increase uptake, adherence and completion of guideline-based cessation treatments among SED smokers who were motivated to attempt quitting. We integrated the SCTNs into a high-traffic community site so that they would become familiar and easily accessible to SED smokers in the course of everyday life.

### Naming the navigator role, hiring “guides”

The research team explored possible job titles through semi-structured interviews with a convenience sample of Head Start parents and staff (*n* = 4). Results suggested that the term “navigator” was unfamiliar and unrecognized in relation to tobacco cessation treatment. Terms like “case worker” and “social worker” had strongly negative connotations. Further, the term “treatment” was not seen as relevant to smoking cessation: Respondents viewed nicotine patches and quitlines as non-medical supports. Based on these findings, we identified navigators as “smoking solutions guides.” We recruited lay candidates rather than professionals to avoid introducing a power differential; candidates were not expected to have counseling training or experience. The team decided to hire two half-time guides rather than a single fulltime person, in order to provide  the guides withpeer support in the face of potentially discouraging interactions (participant no-shows, smoking lapses, intractable social or financial barriers, mental illness issues, *etc.*). Job candidates were recruited at the Head Start site and other community organizations. Required qualifications included bilingual fluency (English and Spanish) and willingness to work half-time. Preferred qualifications included experience working with low-income populations, experience guiding people through a system or health condition, and interest in health promotion.

### Protocol

The SCTN intervention relied on existing community resources for smoking cessation such as state quitlines, free or discounted nicotine replacement therapy (NRT), and first-line cessation prescription medications covered by Medicaid or other programs. We planned to let participants attend up to 12 navigation sessions, expecting participants and guides to tailor individual levels of support across the first several months of the cessation process. The first few sessions were timed to align with cessation treatment milestones and challenges: on or around the quit date; 72 h after quit date (to help manage nicotine withdrawal, assess NRT efficacy, and distinguish withdrawal symptoms from NRT side-effects); then weekly for the first one-to-two months and monthly thereafter. This idealized schedule would be flexible, to accommodate individual needs and the unpredictability of SED life.

In the first session, the navigator helps the participant set a quit date and select an evidence-based cessation strategy – quitline coaching alone or with free NRT; study-provided NRT alone (community option unavailable), or prescription cessation-medication (requires prescriber appointment). During each subsequent session, the navigator assesses self-reported treatment use, identifies and helps overcome barriers and challenges to treatment adherence, and provides motivational reinforcement. If the participant has relapsed, the guide supports consideration of a renewed quit-attempt.

### Training curriculum

Topics include a study overview; basic knowledge of smoking, nicotine dependence, behavioral dependence, and secondhand smoke; the guide role; working with Head Start staff; smoking cessation processes and evidence-based treatments; motivational interviewing; mental health and smoking cessation; relapse prevention, and participant empowerment. Where possible, curricular elements were adapted from the Colorado Patient Navigator Training Program (www.patientnavigatortraining.org). The initial training included 40 h of online and in-person sessions. Problem-based booster sessions consisted of weekly, one-to-two hour meetings where guides and researchers reviewed the week’s sessions, discussed challenges, and identified needs for further skill-building.

### Participant recruitment, eligibility, enrollment

Participants were adult smokers (aged 18+) affiliated with the Head Start study site (parent, grandparent, employee) who were willing to set a quit date within 30 days (initial criterion) or 15 days (revised criterion to emphasize cessation motivation). Daily smoking was not a requirement, because nondaily smoking is more common among Latino populations and because nondaily smokers are especially likely to benefit from cessation interventions. [78] Recruitment occurred in two waves, a year apart (2010-11), during the fall start of the school year. The guides established a sign-up station in the preschool lobby and approached potential enrollees to determine interest and eligibility. Potential participants met with a research assistant to complete the consent process and provide baseline measures. Enrollment occurred when the person attended the first guide session and set a quit date.

### Intervention refinement

The first wave was delivered by two guides; emphasized face-to-face sessions; accepted participants with any level of current smoking; encouraged quit-dates within 30 days, and scheduled follow-up data collection for six months post-enrollment. Modifications for the second wave included: quit-date scheduling within 15 days; follow-up data collection at three months to increase retention for outcome measurement; increased acceptance of telephone sessions; greater accommodation of flexible scheduling; less frequent contact if desired, and greater emphasis on relapse prevention. Only one guide was retained for the second wave, based on mid-study evaluation of MI skills and capacities for empathy with participants.

### Measures

Baseline and follow-up data were collected by self-administered, researcher-reviewed questionnaires and expired carbon monoxide (CO) monitors (Vitalograph Inc., Lenexa, KS). The baseline questionnaire included demographic items, tobacco-related items (history and current status, cessation history, motivation to quit smoking), and self-reported mental health diagnosis (if any). The follow-up questionnaire included primary cessation endpoints (7-day point abstinence, utilization of evidence-based cessation treatment) and satisfaction with the intervention. Expired CO was measured at each collection as the mean of two readings, with mean < 10 ppm considered abstinence. Interim smoking status, cessation treatment utilization, and intervention participation were collected at every guide session.

A semi-structured exit interview explored participant motivations to join the program, experience with quit methods, perceptions of the intervention and types of assistance provided by the guide, reasons for discontinuing the program, and potential program improvements. The exit interview included a card-sort activity in which the participant sorted 21 cards, each bearing a word that theoretically could represent an attribute, into piles of attributes that were “like my guide,” “not like my guide,” or perceived as irrelevant. When the sort was complete, the participant explained why they placed  eachcard where they did.

### Analysis

Descriptive statistics and primary endpoints were calculated using standard techniques for proportions, means, and medians, with non-normal distributions transformed and back-transformed as needed. Outcome rates at follow-up were calculated two ways, available data only and imputed failure for missing data (“intent-to-treat” (ITT) assumption).

Guide sessions were audio-recorded for quality assurance reviews. At study midpoint, 14 sessions (seven per guide) were assessed for MI competency using the Motivational Interviewing Treatment Integrity code (MITI 3.1) [79–81]. The code evaluates both communications style and MI-specific behaviors (adherent, non-adherent); determination of competency is based on four communications dimensions: evocation, collaboration, empathy, and autonomy support. Using a five-point Likert scale, a mean of 3.5 across the four dimensions connotes beginning competency, and 4.0 connotes competency.

Qualitative analysis of recorded interviews proceeded in stages, starting with immersion and creation of broad coding categories based on the interview structure. An iterative process was used to develop initial codes [82]; categories and themes were inductively developed through iterative review of codes [83].

## Results

The study consented 49 individuals (35 in wave 1, 14 in wave 2) and enrolled 40 of them; nine who consented (7 from wave 1, two from wave 2) did not complete the steps for enrollment (attend first guide session, set a quit date).

About two-thirds of enrollees (63 %) were women, two-thirds African American (68 %), and nearly one-fifth Latino (Table 1). Half had more than high school education. One-third self-reported one or more mental health diagnoses: 33 % reported depression, 11 % anxiety, 14 % bipolar and 6 % schizophrenia. At baseline, nearly all enrollees smoked daily (Table 2), a median of 10 cigarettes per day. Most had previously attempted quitting, and half who did had tried NRT; the longest period of abstinence was a mean of 15.8 days.

**Table 1:** Characteristics of pilot study participants

(*n* = 40)	
female	63 %
mean age (CI)	40.1 (32.4, 48.6)
ethnicity*	
black/African American	68 %
Latino	18 %
Anglo/non-Hispanic white	10 %
American Indian	5 %
more than high school education	50 %
health insurance status	
Medicaid	38 %
other (includes indigent care program)	29 %
none or status unknown	33 %
sole adult in household	43 %
married/living as married	35 %
employment status*	
wage-worker or self-employed	38 %
disabled	25 %
student	15 %
unemployed	10 %
stay-at-home parent	5 %
retired	5 %
mental diagnosis (self-reported, may report >1 type)	
depression	33 %
bipolar	14 %
anxiety	11 %
schizophrenia	6 %

CI: 95 % confidence interval

*does not sum to 100 % due to rounding

**Table 2:** Smoking and cessation history

(*n* = 40)	
currently smoke daily	98 %
median cigarettes per day	10
first cigarette ≤30 min of waking	68 %
mean baseline CO (ppm)	17.5
level indicated abstinence*	23 %
any lifetime quit attempt	80 %
median number of quit attempts	2
median days (CI) of longest abstinence	9 (4, 30)
ever used NRT (if any quit attempt)	52 %
ever used quitline (if any quit attempt)	39 %
past year quit attempt	60 %
past year provider advice to quit	
yes	60 %
no	15 %
did not see provider	13 %
no answer	13 %
how confident can stay quit one month (1-10 scale)	6.1

CO = expired carbon monoxide; NRT = nicotine replacement therapy

*< 10 ppm

The two guides diverged on MI competency. One was rated beginning competent with a mean score of 3.65 while the other scored 2.8, well below the cutoff of 3.5. The main difference was on the empathy dimension, where the below-proficient guide scored 2.3.

Follow-up data were obtained from 34 participants (85 %). Most had made a quit-attempt and initiated evidence-based treatment during the study (82 %; ITT = 70 %; Table 3). Seven (21 %; ITT = 18 %) self-reported seven-day point-abstinence, of whom three had confirming CO levels. Among abstinent and relapsed participants combined, the longest period of abstinence was a mean of 27.6 days. Among relapsed participants, 70 % were highly motivated to make another quit-attempt (≥9 on 1-10 scale). Outcomes did not differ significantly between study waves (data not shown), although mean months to follow-up was longer in wave 1 than in wave 2 (5.8 vs. 3.6, p < 0.0001). Participants attended a mean of 3.4 sessions (mean duration, 30.0 min). Guides were highly rated on all queried attributes, with mean scores of 5.1 to 6.2 (1-7 scale, 1 = “strongly disagree,” 7 = “strongly agree”).

**Table 3:** Study outcomes

Cessation behaviors and outcomes		
provided follow-up data	85 % (*n* = 34/40)	
mean time to follow-up (months (CI))	5.2 (4.7, 5.7)	ITT*
made a quit attempt	82 %	70 %
initiated treatment	82 %	70 %
enrolled in quitline (QL)	50 %	43 %
completed all QL sessions (if enrolled)	28 %	–
used NRT	71 %	60 %
self-reported abstinence (≥7 days)	21 %	18 %
CO-confirmed abstinence†	9 %	–
median days (CI) longest abstinence during study (if >0)	21 (7, 58)	–
relapsed, highly motivated to try again (≥9 on 10-point scale)	70 %	–
program utilization and satisfaction		
mean number (CI) of guide sessions completed	3.4 (2.5, 4.2)	
mean duration (CI) of guide sessions (minutes)	30.0 (27.0, 32.6)	
“very likely” to recommend guide program to family, friend	74 %	63 %
time with guide was “about the right amount”	68 %	58 %
guide’s advice was “very helpful”	67 %	55 %
program increase chance of success “a lot”	59 %	50 %
sessions increased quit-confidence “a lot”	56 %	48 %
sessions helped with problems in quitting	44 %	38 %
program was “very helpful” (vs. “somewhat” or “not very”)	41 %	35 %
“very hard” to fit guide sessions into schedule	35 %	45 %
guide attributes (1-7 scale)	mean	median
responsive	6.2	7
listened	6.2	7
supportive	6.1	7
trusted	5.9	6
collaborative	5.5	6
directive	5.4	6
helpful	5.3	6
important to my quitting	5.1	6
empathetic	5.1	6

CI = 95 % confidence interval

*intent-to-treat analysis: no follow-up ≡ failed outcome

† < 10 ppm among participants self-reporting 7-day point abstinence

Analysis of exit interviews identified seven emergent themes (Table 4). Trust: Participants felt safe to discuss highly personal information. This openness also required guides to manage interactions efficiently, maintain focus, and be clear about professional boundaries; participants generally resisted any suggestion of referral for mental health counseling. Autonomy: Most participants reported feeling supported to make their own choices. Convenience: Meeting at the Head Start site and over the phone  werecited as very convenient since participants were regularly dropping off and picking up their children. Coaching: Participants often referred to guides as coaches, or someone on the sidelines who provided emotional support and training and was watching the person succeed. Education: All participants said the guides provided information to assist them during the cessation process. Confidence: Many participants said the guides provided external confidence. Instrumental support: Many participants cited tangible assistance such as provision of NRT, help calling the quitline, and looking up information.

**Table 4:** Illustrated themes from participant interviews about the *Smoking Solutions Guide* program

Theme	Participant quotes
trust	“I trusted her and could tell her anything.”
	“I didn’t feel like she went out of this room and talked about me.”
autonomy	“The guide asked me what I wanted to do – she didn’t push.”
convenience	“After my meeting (with the guide) I could pick up my kids and go home. It was a good place to come to.”
coach	“She helped coach me through the difficult times. When I needed her the most, she was the only one to support me with my quitting. She was not judgmental. She was someone so supportive I could go to her.”
education	“We discussed triggers and cravings. I didn’t know anything about that.”
confidence	“She was like my cheering section. When I thought I couldn’t do it, she was always there.”
instrumental support	“When I didn’t have patches, they gave me patches and also lozenges. Financial-wise it helped, because I couldn’t afford it, and they always had back up for me.”
	“I didn’t know how to go to my health provider – I didn’t know what to say – so she helped me [figure out] what questions to ask before.”

**Fig. 1:**
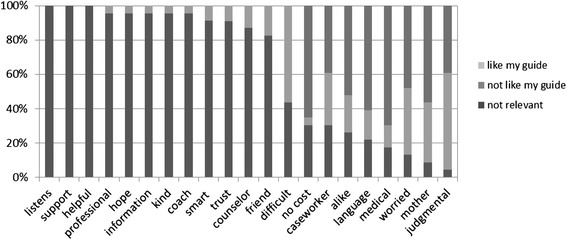
How exiting participants (n = 23) rated their guides on prompted attributes and behaviors

In the card-sorting activity, most participants (≥90 %) identified each of 10 words with their guides: listens, support, helpful, professional, hope, information, kind, coach, smart, and trust (Fig. 1). Few participants saw the guides as medical, and few saw them as like themselves. Many participants expressed strong negative reactions to the word *caseworker*, which was viewed as a dehumanizing role, one who does not see the client as a person but as a case or number, with little concern for the person.

## Discussion

A pilot study among low-income smokers found that community-based *smoking solutions guides* were very well accepted and successful in connecting participant smokers to evidence-based cessation resources. Quit-related outcomes were promising, with 70 % of participants making attempts and 18 % reporting 7-day abstinence at three- to six-month follow-up (intent-to-treat rates). Quit-attempters remained abstinent longer on average than in previous attempts, although the  differencewas not significant. Overall, the results support continued research under rigorous evaluation designs.

The study found promising rates of program utilization and research retention. A primary driver may have been location: The program was integrated into an early-learning center that program participants visited daily to drop off and pick up their children. Explicit research attention could focus on the potential of such well-traveled “commuter” settings to reach and engage SED smokers, since such settings could extend the reach of evidence-based treatments and help normalize quitting for smokers who do not view cessation as a medical matter.

Satisfaction with the guides and the program was widespread, and majorities of participants said the program helped them “a lot” and increased their chances of successfully quitting. At the same time, more than one-third found it “very hard” to fit guide sessions into their schedules. Future studies will need to explore ways to reduce this barrier, such as shorter sessions and alternative contact modes.

The two study guides differed in MI competency after training, and one was not retained for the second wave of the study because of difficulty empathizing with SED participants. Hiring interviews focused on inter-personal skills, problem-solving, ability to work independently and experience working with SED communities. One guide’s difficulty with empathy suggests a need for our intervention to improve both pre-hiring assessment and post-hiring enhancement of empathy skills. Guides must understand appropriate and effective personal disclosure to support relationship-building. Refraining from judgment is essential and may not be assumed as a non-professional norm. Given the complexity and difficulty of health behavior change, compounded by nicotine dependence, and further compounded by chronic and acute stressors of SED life, guides must not only refrain from expressing judgment but must not tacitly feel that struggling participants are “non-compliant” or “unwilling.”

Effective guides must also balance good boundaries with demonstration of interest and commitment to participants’ wellbeing. Hiring interviews should assess willingness to refrain from advice and rescue in favor of joining with a person’s own problem-solving activity. Finding such candidates is a first step but also requires training and reinforcement, since helping participants find their own solutions requires considerable skill and may be perceived as time-consuming.

Participants clearly appreciated initial contacts in person to establish rapport, and most expressed appreciation for their guides’ dedication of time to support their smoking cessation effort. At the same time, participants generally chose to have subsequent sessions occur by telephone. A previous study of telephone vs. in-person cessation treatment found that treatment modality was unrelated to long-term abstinence, but in-person treatment was more effective in early abstinence [84].

Throughout the guide sessions, participants brought up a variety of acute and chronic stressors, ranging from balancing of work/school/ parenting responsibilities to having a son sent to jail. Conventional approaches to relapse prevention may be inadequate in the face of socioeconomic hardship and racial discrimination. In a review of socioeconomic status and smoking, Hiscock *et al.* posit that life stress falls outside the purview of smoking cessation programming. We respectfully disagree and suggest that the acquisition of alternative coping strategies, like NRT for highly dependent smokers [24], may be essential for SED smokers to organize and manage smoking cessation processes in the face of chronic hardships. Appropriately trained guides may be able to assess stressors and help SED smokers acquire or enhance coping strategies other than cigarettes, a possibility the authors are currently starting to explore in pilot research.

Study development and implementation generated numerous formative lessons, ranging from the merely suggestive to the potentially transferable. Among the more persuasive findings is the apparent potential to engage SED smokers in treatment when guides are integrated into well-traveled community settings. The study readily found and engaged smokers who were ready to try quitting and open to using evidence-based treatment. SED populations generally under-utilize evidence-based cessation treatment [22, 25, 26, 84–87], although participants in the current study reported rates of previous NRT use that are comparable to general population rates. Cost may generally be a barrier to NRT use among SED populations, but cost-elimination studies have produced mixed results, with some finding little change in uptake [88, 89] or outcome [90, 91] when cost was eliminated. We speculate that face-to-face contact with a friendly, non-threatening guide may serve as a psychosocial bridge to cessation treatment.

Some limitations apply to the current study. It was designed to assess feasibility and thus did not support evaluation of intervention efficacy. Participants were parents, other family members and staff from a single Head Start site, and results may not apply to other Head Start sites, preschool settings in general, or other community settings. Prevalence of mental health diagnoses was based on self-report and may over- or underestimate true rates. The authors are currently developing proposals to address these limitations using rigorous and more generalizable study designs. If community-based smoking solutions guides prove both efficacious and engaging, initiatives to deploy them across communities will likely cost more than reliance on quitlines as the primary public health strategy for smoking cessation. But given quitlines’ limited reach, and the growing segregation of highly prevalent smoking to SED populations, allocation of additional resources may be the only way to revive progress toward the end of the U.S. cigarette epidemic.

## Conclusions

A pilot study found that community-based guides may increase uptake of evidence-based smoking cessation treatment among socioeconomically disadvantaged smokers. More rigorous study is warranted.
